# Assessment and an updated list of the mosquitoes of Saudi Arabia

**DOI:** 10.1186/s13071-019-3579-4

**Published:** 2019-07-19

**Authors:** Azzam M. Alahmed, Kashif Munawar, Sayed M. S. Khalil, Ralph E. Harbach

**Affiliations:** 1 Plant Protection Department, College of Food and Agricultural Sciences, King Saud University, Riyadh, Saudi Arabia; 2 Agricultural Genetic Engineering Research Institute, Agricultural Research Center, 9 Gamaa Street, Giza, Egypt; 3 Department of Life Sciences, Natural History Museum, Cromwell Road, London, SW7 5BD UK

**Keywords:** Culicidae, Mosquitoes, Saudi Arabia, Vectors

## Abstract

**Background:**

Mosquito-borne pathogens are important causes of diseases in the Kingdom of Saudi Arabia. Knowledge of the mosquito fauna is needed for the appropriate control of the vectors that transmit the pathogens and prevent the diseases they cause. An important first step is to have an up-to-date list of the species known to be present in the country. Original occurrence records were obtained from published literature and critically scrutinized to compile a list of the mosquito species that occur within the borders of the Kingdom.

**Results:**

Fifty-one species have been recorded in the Kingdom; however, the occurrence of two of these species is unlikely. Thus, the mosquito fauna of the Kingdom comprises 49 species that include 18 anophelines and 31 culicines. Published records are provided for each species. Problematic records based on misidentifications and inappropriate sources are discussed and annotated for clarity.

**Conclusion:**

Integrated morphological and molecular methods of identification are needed to refine the list of species and accurately document their distributions in the Kingdom.

## Background

The Arabian Peninsula (*c.*3 million km^2^) includes the Kingdom of Saudi Arabia (KSA), Oman, Qatar, United Arab Emirates and Yemen. The KSA occupies the major portion of the peninsula (*c.*1.97 m km^2^), and includes 13 provinces. Mosquito-borne pathogens cause several diseases in the KSA. Malaria has been considered an endemic disease in the country from as early as 1900 [1].

Many studies have focused on the identification and ecology of the mosquito fauna of the peninsula, including the KSA [2–7]. The distribution and abundance of mosquito species are influenced by host availability and human activities, changes in land cover and climatic conditions, such as temperature and rainfall [8]. These conditions significantly affect the vectorial capacity of anopheline and other mosquitoes for pathogen transmission. Understanding these factors is essential for disease control.

Mosquito-borne pathogens, including *Plasmodium* species, dengue virus, Rift Valley fever virus and microfilariae, cause diseases in the KSA [9–11]. Correct identification of vector species and knowledge of their distributions and biology are important requirements for disease control.

Historically, mosquitoes have been collected in different areas of the KSA during ecological studies and for construction of morphological keys for their identification. Prior to 1956 [2], little was known about the mosquito fauna of the Arabian Peninsula, with virtually nothing known about mosquitoes in the central and northern regions of the peninsula. Records of mosquito species collected in the KSA are found principally in papers published before the end of the last century [2, 4, 5, 12–14]. Additional species have been recorded in more recent publications, but until now a list of all species of mosquitoes present in the country has not been published. Therefore, the purpose of this work was to produce an up-to-date list of the mosquito species that are definitely known to occur in the KSA, annotated with published collection records.

## Methods

PubMed, ResearchGate, Saudi Digital Library and Scopus were searched for articles on the Culicidae of Saudi Arabia using the terms “mosquito” and “Saudi Arabia” separately and in combination. Articles recovered from the search were used as a source of possible additional articles. All references obtained from the searches were examined for species and collection records, and the data were recorded in a spread sheet. The data were used to compile an up-to-date list of the mosquito species that are unequivocally known to occur in the KSA. The abbreviations for genera in the S3 Appendix of Wilkerson et al. [15] are used herein.

## Results

Search of the literature revealed that 51 mosquito species have been found in the KSA from 1956 to 2017. However, the occurrence of two of these species in the country is doubtful (see “Discussion”), leaving 49 species (18 anophelines and 31 culicines). The anophelines include proven or suspected vectors of malarial protozoa and the culicines include proven or suspected vectors of dengue fever, chikungunya, Zika, yellow fever, West Nile and Rift Valley fever viruses, as well as microfilariae. The 49 species are listed below. The listing is arranged alphabetically by genus, subgenus and species. The aedine species are listed as species of *Aedes* in accordance with the classification of Wilkerson et al. [15]; anopheline and non-aedine species are listed under the generally accepted genera and subgenera recognized in the online Mosquito Taxonomic Inventory (http://mosquito-taxonomic-inventory.info/). Published records are listed for each species, some with brief notes for clarification.

### List of the mosquito species known to occur in the KSA

**Genus** ***Aedes*** **Meigen, 1818**

**Subgenus** ***Aedimorphus*** **Theobald, 1903**

1. *Ae.* (*Aedimorphus*) *vexans* (Patton, 1905)

Records: [2] (as *Ae. arabiensis*); [6, 16] (as *Aedimorphus v. arabiensis*), [17–23] (as *Aedimorphus arabiensis*); [24, 25]; [26] (as *Ae. vexans*).

**Subgenus** ***Fredwardsius*** **Reinert, 2000**

2. *Ae.* (*Fredwardsius*) *vittatus* (Bigot, 1861)

Records: [6, 16, 23] (as *Fredwardsius vittatus*); [17] (as species of subgenus *Aedimorphus*); [18, 21, 24, 25, 27, 28].

**Subgenus** ***Ochlerotatus*** **Lynch Arribálzaga, 1891**

3. *Ae.* (*Ochlerotatus*) *caballus* (Theobald, 1912)

Records: [17] (as *Ochlerotatus caballus*); [24]; [27].

4. *Ae.* (*Ochlerotatus*) *detritus* (Haliday, 1833)

Records: [6, 7] (as *Ochlerotatus detritus*).

5. *Ae.* (*Ochlerotatus*) *caspius* (Pallas, 1771)

Records: [2, 18–20, 24–26, 28–40]; [6, 7, 16, 17] (as *Ochlerotatus caspius*).

**Subgenus** ***Stegomyia*** **Theobald, 1901**

6. *Ae.* (*Stegomyia*) *aegypti* (Linnaeus, 1762)

Records: [2, 18, 20, 21, 24, 25, 27, 28, 30, 34, 41–49]; [6, 7, 16, 23, 26] (as *Stegomyia aegypti*).

7. *Ae.* (*Stegomyia*) *unilineatus* (Theobald, 1906)

Records: [21, 24, 50].

**Genus** ***Anopheles*** **Meigen, 1818**

**Subgenus** ***Anopheles*** **Meigen, 1818**

8. *An.* (*Anopheles*) *coustani* Laveran, 1900

Records: [2, 3, 14, 51–54].

9. *An.* (*Anopheles*) *tenebrosus* Dönitz, 1902

Records: [1, 6, 14, 24, 29, 31, 35]; [2, 55] (as *An*. *coustani* var. *tenebrosus*).

**Subgenus** ***Cellia*** **Theobald, 1902**

10. *An.* (*Cellia*) *arabiensis* Patton, 1905

Records: [1, 16, 22, 26, 56–59]; [2, 6, 30, 35, 51] (as *An*. *gambiae*); [3, 24] (as *An. arabiensis* and *An. gambiae* (*s.l.*)); [7, 13, 14, 27, 29]; [12, 60] (as *An*. *gambiae* species B); [61, 62] (as *An*. *gambiae* (*s.l.*)).

11. *An.* (*Cellia*) *azaniae* Bailly-Choumara, 1960

Records: [7, 17, 18, 24, 32].

12. *An.* (*Cellia*) *cinereus* Theobald, 1901

Records: [2, 3, 6, 7, 14, 16, 18–20, 24, 28, 34, 35, 59, 61].

13. *An.* (*Cellia*) *culicifacies* Giles, 1901 (*s.l.*)

Records: [3, 28] (as *An. culicifacies adenensis*); [6, 24, 61]; [13] (as *An. culicifacies* probably species A); [16] (as *An. culicifacies* (*s.l.*)).

14. *An.* (*Cellia*) *dthali* Patton, 1905

Records: [1, 3, 18, 20, 26, 28, 30, 34, 35, 41, 44, 57] (as *An. d’thali*); [2, 6, 7, 14, 16, 21, 22, 24, 27, 32, 39, 51, 56, 59, 61]; [19] (as *An. d<thali*); [33] (as *An. azaniae*? and *An. d’thali*).

15. *An.* (*Cellia*) *fluviatilis* James, 1902 (*s.l.*)

Records: [1, 2, 12, 14, 24, 26, 31, 35, 37, 51, 55, 57].

16. *An.* (*Cellia*) *multicolor* Cambouliu, 1902

Records: [1–3, 6, 7, 14, 16, 19, 22, 24, 27–30, 32–36, 40, 41, 51, 55–57].

17. *An.* (*Cellia*) *pharoensis* Theobald, 1901

Records: [2, 3, 14]; [51] (misspelled as *phorensis*).

18. *An.* (*Cellia*) *pretoriensis* (Theobald, 1903)

Records: [3, 6, 14, 18, 24, 26–29, 32, 35, 39, 56, 58].

19. *An.* (*Cellia*) *pulcherrimus* Theobald, 1901

Records: [2, 12, 14, 24, 55].

20. *An.* (*Cellia*) *rhodesiensis rupicolus* Lewis, 1937

Records: [1, 3]; [14, 24, 56, 59] (as *rupicola*); [22, 27] (as *An. rupicolus*); [28, 57]; [30, 35] (as *An. rhodesiensis*); [33] (as *An. azaniae*? and *An. rhodesiensis*); [51] (as *An. rhodesiensis*, misspelled as *rohesiensis*).

21. *An.* (*Cellia*) *sergentii* (Theobald, 1907)

Records: [1–3, 6, 12–14, 16, 22, 24, 27, 29, 31, 35, 55–57, 61, 62]; [7, 30, 37, 51] (as *sergenti*).

22. *An.* (*Cellia*) *stephensi* Liston, 1901

Records: [1, 2, 6, 12–14, 18–20, 23, 24, 28, 30, 32–35, 37, 39, 51, 55, 59].

23. *An.* (*Cellia*) *subpictus* Grassi, 1899 (*s.l.*)

Records: [6, 20, 24, 28, 30, 32, 33, 35, 51, 61].

24. *An.* (*Cellia*) *superpictus* Grassi, 1899

Records: [1, 7, 12, 14, 19, 20, 28, 34, 35].

25. *An.* (*Cellia*) *turkhudi* Liston, 1901

Records: [1–3, 6, 7, 14, 16, 20, 22, 24, 26–28, 30, 32, 33, 51, 56, 57, 61].

**Genus** ***Culex*** **Linnaeus, 1758**

**Subgenus** ***Barraudius*** **Edwards, 1921**

26. *Cx.* (*Barraudius*) *pusillus* Macquart, 1850

Records: [2, 6, 24, 32, 34–36, 40].

**Subgenus** ***Culex*** **Linnaeus, 1758**

27. *Cx.* (*Culex*) *decens* Theobald, 1901

Records: [16, 24, 26, 63]; [64] (requires confirmation).

28. *Cx.* (*Culex*) *duttoni* Theobald, 1901

Records: [7, 16, 22, 24, 63]; [64] (requires confirmation).

29. *Cx.* (*Culex*) *laticinctus* Edwards, 1913

Records: [2, 4–7, 16, 18–20, 24, 28, 30, 32, 34, 35, 39, 64].

30. *Cx.* (*Culex*) *mattinglyi* Knight, 1953

Records: [4–6, 16, 24, 32].

31. *Cx.* (*Culex*) *mimeticus* Noè, 1899

Records: [4, 7, 16, 24].

32. *Cx.* (*Culex*) *perexiguus* Theobald, 1903

Records: [4, 5, 16, 20, 24, 28, 36, 40, 41]; [6, 19, 32, 33, 35, 39] (as *Cx. perexiguus* and *Cx. univittatus*); [31, 34, 64] (as *Cx. univittatus*).

33. *Cx.* (*Culex*) *pipiens* Linnaeus, 1758

Records: [2] (as *Cx*. *pipiens* and *Cx*. *pipiens* var. *molestus*); [4, 5, 24, 65]; [6] (undoubtedly includes *Cx. quinquefasciatus* and hybrids); [7] (may be misidentification of *Cx. quinquefasciatus*); [19, 34, 64] (*Cx. pipiens* and *Cx. quinquefasciatus* = hybrids?); [20, 28, 53] (may be or include *Cx. quinquefasciatus*); [23] (probably includes *Cx. quinquefasciatus*); [26] (may be misidentification of *Cx. quinquefasciatus*); [30, 33, 36, 40, 44] (probably hybrids); [31] (as *Cx. pipiens* complex = hybrids?); [35] (probably includes hybrids?); [37] (as *Cx. molestus*); [41, 66] (northern west coastal localities only); [67] (as *Cx. pipiens* and *Cx. quinquefasciatus*, probably includes hybrids).

34. *Cx.* (*Culex*) *quinquefasciatus* Say, 1823

Records: [2] (as *Cx*. *pipiens* ssp. *fatigans*); [4, 5, 7, 24, 26, 28, 31, 65]; [6, 30, 38] (probably includes hybrids); [16] (as *Cx. pipiens* and *Cx. quinquefasciatus*); [17, 21, 22] (as *Cx. pipiens* complex); [18] (may be or include *Cx. pipiens*); [19, 29] (as *Cx. pipiens* = hybrids? and *Cx. quinquefasciatus*); [20, 23] (may include *Cx. pipiens*); [32, 39] (as *Cx. pipiens* and *Cx. quinquefasciatus*, probably includes hybrids); [33] (may include hybrids); [34, 64] (probably hybrids); [35] (in southern areas of eastern region); [41, 66] (mid-west coastal area only, probably includes hybrids); [44] (*Cx. pipiens* = hybrids and *Cx. quinquefasciatus*).

35. *Cx.* (*Culex*) *simpsoni* Theobald, 1905

Records: [6] (southwest only); [16, 18] (may be or include *Cx. sinaiticus*); [24, 28].

36. *Cx.* (*Culex*) *sinaiticus* Kirkpatrick, 1925

Records: [4, 5, 20, 22, 24, 28, 30, 64]; [16, 18, 68] (may be or include *Cx. simpsoni*); [26] (may include or be misidentification of *Cx. simpsoni*); [32, 39] (as *Cx. perexiguus* and *Cx. simpsoni*); [33, 35] (as *Cx. simpsoni*); [61] (misidentification as *Cx. simpsoni* in areas north of southwest).

37. *Cx.* (*Culex*) *sitiens* Wiedemann, 1828

Records: [2, 4, 5, 18, 22, 24, 26, 28, 41, 61].

38. *Cx.* (*Culex*) *theileri* Theobald, 1903

Records: [4, 5, 7, 16, 20, 24, 28, 29, 32, 33, 36, 37, 39, 41].

39. *Cx.* (*Culex*) *tritaeniorhynchus* Giles, 1901

Records: [4, 5, 7, 16–24, 26, 28, 30–36, 39, 41, 44, 45, 68].

40. *Cx.* (*Culex*) *univittatus* Theobald, 1901

Records: [18] (may be or include *Cx. perexiguus*)]; [20] (requires confirmation, might be *Cx. perexiguus*); [24, 28]; [30] (probably *Cx. perexiguus*); [36] (undoubtedly includes *Cx. perexiguus*).

**Subgenus** ***Culiciomyia*** **Theobald, 1907**

41. *Cx.* (*Culiciomyia*) *nebulosus* Theobald, 1901

Records: [16, 21, 24, 37].

**Subgenus** ***Maillotia*** **Theobald, 1907**

42. *Cx.* (*Maillotia*) *arbieeni* Salem, 1938

Records: [7, 16, 28, 30, 36].

43. *Cx.* (*Maillotia*) *salisburiensis* Theobald, 1901

Records: [16, 21, 24].

**Subgenus** ***Oculeomyia*** **Theobald, 1907**

44. *Cx.* (*Oculeomyia*) *bitaeniorhynchus* Giles, 1901

Records: [16, 24, 26, 63].

**Genus** ***Culiseta*** **Felt, 1894**

**Subgenus** ***Allotheobaldia*** **Brolemann, 1919**

45. *Cs.* (*Allotheobaldia*) *longiareolata* (Macquart, 1838)

Records: [2, 16, 18–20, 23, 24, 26, 28, 30–36, 39–41].

**Subgenus** ***Culiseta*** **Felt, 1894**

46. *Cs.* (*Culiseta*) *subochrea* (Edwards, 1921)

Records: [24] (as *Cs. annulata* and *Cs. subochrea*); [29, 36, 40]; [31] (as *ochracea annulata*).

**Genus** ***Lutzia*** **Theobald, 1903**

**Subgenus** ***Metalutzia*** **Tanaka, 2003**

47. *Lt.* (*Metalutzia*) *tigripes* (de Grandpre & de Charmoy, 1901)

Records: [2, 18–20, 22, 30] (as *Cx. tigripes*); [7, 16, 24, 26, 36].

**Genus** ***Coquillettidia*** **Dyar, 1905**

48. *Cq.* (*Coquillettidia*) *richiardii* (Ficalbi, 1889)?

Record: [31] (as *Mansonia* sp.).

**Genus** ***Uranotaenia*** **Lynch Arribálzaga, 1891**

**Subgenus** ***Uranotaenia*** **Lynch Arribálzaga, 1891**

49. *Ur*. (*Uranotaenia*) *unguiculata pefflyi* Stone, 1961

Records: [7, 24, 31, 35] (as *Ur. unguiculata*); [69].

## Discussion

The ecological diversity of the KSA allows multiple opportunities for the evolution of variation in both mosquitoes and their pathogens. Large-scale movements of people, including the annual pilgrimage to Mecca, large-scale use of foreign workers and new developmental projects engender environmental heterogeneity and complex epidemiology. The wide variation in ecological settings may complicate the understanding of spatial/temporal characteristics and population dynamics of mosquito vectors of pathogens that cause diseases in humans.

Investigators may be reluctant to conduct ecological studies in the KSA because field work is labour-intensive and time-consuming. Moreover, due to habitat heterogeneity, which in the KSA is often extreme, there are species of only seven mosquito genera in the country. Here we present the first complete list (see above) of the mosquito species that definitely are known to occur in the KSA. In some cases, ecological and morphological studies have shown that some species are members of species complexes. There are members of four *Anopheles* species complexes in the country, including the *An. gambiae*, *An*. *fluviatilis*, *An*. *culicifacies* and *An*. *subpictus* complexes. The Afrotropical *An. gambiae* complex consists of eight species, which includes malaria vectors (*An. gambiae*, *An. arabiensis*, *An. bwambae*, *An*. *coluzzii*, *An. merus* and *An. melas*) and non-vectors (*An. quadriannulatus* and *An. amharicus*) [70, 71]. *Anopheles arabiensis* is the only member of the complex with a range that extends outside of Africa into the southwestern and western areas of the Arabian Peninsula, occurring in the KSA and Yemen, where it is the major malaria vector [2, 3, 7, 72–75]. Most ecological studies conducted in the KSA recorded the presence of *An. arabiensis* (often as *An. gambiae*) in different life stages [27, 29, 30, 51]. Moreover, molecular analysis based on the internal transcribed spacer (ITS2) region of rDNA has shown that *An. arabiensis* (GenBank: KM068071) is the only member of *An. gambiae* complex present in the KSA [59].

The *An. fluviatilis* complex is a group of mosquitoes that is widely distributed in southern and southwestern Asia and includes important malaria vectors [76]. The complex includes four species informally denoted as species S, T, U and V [77–81]. *Anopheles fluviatilis* S is mainly anthropophilic, endophilic and a very competent malaria vector [52]. Species T and U are primarily zoophilic, exophagic and exophilic, and are regarded as poor- or non-vectors of malaria even though they are responsible for malaria transmission in mountainous and hilly areas in Asia, including Iran [80, 82]. *Anopheles fluviatilis* (*s.l*.) was recorded in the eastern and western regions of the KSA by Mattingly & Knight [2], Wills et al. [31] and Al-Ghamdi et al. [51]. It is not known whether species T or U or both are present in the KSA.

The *An. culicifacies* complex consists of five sibling species, informally named species A, B, C, D and E, based on cytotaxonomic and molecular evidence [53, 76, 83]. These species have distinct geographical distributions, blood-feeding behaviour and vectorial capacity for malaria transmission [54, 84, 85]. The complex is widely distributed from southern China to Iran, the Arabian Peninsula and Ethiopia [86]. Species E is the most important malaria vector in the Indian subcontinent, and is highly anthropophilic and endophilic [87]. The other sibling species have low anthropophilic index or are highly zoophilic, and are considered poor- or non-malaria vectors in India and Sri Lanka [52, 88]. Available data indicate there are at least two species of the complex in Iran [89], but only the presence of species A has been confirmed based on cytogenetic and molecular evidence; the second species may be a new member of the complex [90]. Further study is needed to determine which member or members of the complex are present in the KSA.

The *An. subpictus* complex consists of four species in India, informally denoted as species A, B, C and D, based on morphological, chromosomal and molecular differences [91, 92]. As is the case in Iran [90], it is not known which species of the complex is present in the KSA.

*Anopheles stephensi* is the main malaria vector in the eastern region of the KSA, but members of the *An. fluviatilis* and *An. culicifacies* complexes, which are important malaria vectors in other parts of Asia [2, 3, 55, 74], may contribute to malaria transmission. In Asia, *An. stephensi*, *An. culicifacies* (*s.l*.) and *An. fluviatilis* (*s.l*.) consist of different ecoforms that are restricted to rural or urban areas with different degrees of involvement in malaria transmission [54, 76, 85]. *Anopheles stephensi* is a major vector in Asia and the Middle East, including, in addition to the eastern KSA, areas of Iran, Pakistan, Afghanistan, India and southern China [87]. It includes three ecological variants (egg phenotypes): the *mysorensis*, typical and intermediate forms, which are based and distinguished on egg morphology [87, 93–96]. The typical (type) form is mainly found in urban areas, whereas the intermediate and *mysorensis* forms are mainly found in rural environments [87]. The blood-feeding behaviour and role of these forms in malaria transmission varies in different geographical regions. In Iran, the type form is highly anthropophilic and a major malaria vector, whereas the *mysorensis* form is mainly zoophilic and a poor- or non-vector [97]. In contrast, the *mysorensis* form is an important malaria vector in India [98]. These differences might be due to the type of host available and the presence of certain mosquito genotypes with different host preference and susceptibility to malarial parasites. Molecular study using two different markers (ITS2 and *cox*1) did not reveal any differences between *An. stephensi* (ITS2, GenBank: KM052589; and *cox*1, GenBank: KJ528887–KJ528894) in two distant cities of the KSA (Al-Ahsaa and Dammam) where different phenotypes might be present [59].

*Anopheles azaniae* was first encountered in Saudi Arabia during the collection of mosquitoes for Rift Valley virus isolation in the Jizan Region [17], and subsequently collected in the same region by Alahmed et al. [18] and Khater et al. [7]. The record of this species in the Riyadh Region [32], which is a very large eastern region that shares its southern border with Yemen, requires confirmation. The record of this species in the more northerly western Al-Madinah Al-Munawarah Region [33] is doubtful and a probable misidentification of either *An. dthali* or *An. rhodesiensis rupicolus*. *Anopheles azaniae* was previously known to occur in neighbouring Yemen [14], where two morphological forms were recently discovered, the typical form and a previously unrecognized form not immediately identifiable as *An. azaniae* [99]. A single specimen of the atypical form had a mixed human/bovine blood meal, indicating that females are anthropophilic and should be considered a potential vector of malarial protozoa. It seems possible that further studies in the southwest of the KSA may detect the presence of the atypical form.

A species of the genus *Coquillettidia* collected in the Eastern Province of the KSA was identified as a species of *Mansonia* in the study of Wills et al. [31]. According to the online catalogue of the Walter Reed Biosystematics Unit [24], only two species of the tribe Mansoniini are known to occur in more or less arid countries of the Middle East, *Cq*. (*Coquillettidia*) *buxtoni* in Israel and Syria and *Cq*. (*Coq*.) *richiardii* in Iran, Syria and Turkey. With that in mind, it seems likely that the generic identification of Wills et al. [31] was based on keys available at the time, e.g. Mattingly [100], which treated *Coquillettidia* as a subgenus of *Mansonia*. In view of the proximity of eastern areas of the KSA to Iran, we are confident that the mosquitoes identified as a species of *Mansonia* by Wills et al. [31] were most likely specimens of *Cq. richiardii*.

Many of the occurrence records for *Cx. pipiens* and *Cx. quinquefasciatus* in the KSA are problematic. *Culex pipiens* generally occurs north of a line transecting the KSA between Jeddah in the west and Al-Hasa in the east. *Culex quinquefasciatus* occurs in the more southerly region of the Arabian Peninsula, with isolated populations of *Cx. pipiens* occurring in the southwestern mountains [5]. Introgression occurs between the two species where their distributions overlap in central KSA (see figure 3 in Harbach [101]). Both species exhibit considerable variation in the larval and adult stages, and they cannot always be distinguished with certainty. They are only reliably distinguished by characters of the male genitalia; however, their separation is complicated by the occurrence of hybrid forms in the central transitional zone and eastern coastal region of the country. Hybrids are morphologically more variable than the parental species, and are only reliably distinguished from the parental species by the DV/D ratio calculated from measurements based on the form and relationships of the dorsal and ventral arms of the male genitalia [102, 103]. It is not possible to distinguish hybrid larvae and females from the parental species. For this reason, some hybrid specimens collected in and around the zone of introgression have undoubtedly been misidentified as either *Cx. pipiens* or *Cx. quinquefasciatus*. Larvae and adults collected north of the hybrid zone that have been identified as *Cx. quinquefasciatus* are misidentified specimens of *Cx. pipiens*, and those collected south of the zone that have been identified as *Cx. pipiens*, with the possible exception of those collected in the southwestern mountains, are misidentified specimens of *Cx. quinquefasciatus*. Future studies based on dissections of male genitalia or DNA sequence analysis of larvae and adults are needed to substantiate species identifications and collection records. *Culex quinquefasciatus* is an important vector of the microfilariae that cause lymphatic filariasis.

*Culex torrentium* is listed as a species of the KSA in the online catalogue of the Walter Reed Biosystematics Unit [24] based on a number of reports, including Alahmed et al. [18, 19], Al Ahmed et al. [20, 28], Kheir et al. [34], Alahmed [35] and Hassan et al. [41]. However, it is obvious that the identification of *Cx. torrentium* in the KSA by these authors is based on unreliable characters of larvae and adult habitus principally using the keys of Harbach [4, 5]. This species can only be distinguished from members of the *Cx. pipiens* complex with certainty based on features of the male genitalia. Additionally, *Cx. torrentium* is a Palaearctic species whose distribution only extends south into Turkey and possibly into northern Iran [90, 104, 105]. As noted by Harbach [5], early records of *Cx. torrentium* in Iraq are doubtful, and the species is not now listed as present in that country [106, 107]. For these reasons, *Cx. torrentium* is excluded from the list of mosquito species known to occur in the KSA.

Four species of the subgenus *Culex* of the genus *Culex* occur in the southwestern Afrotropical Region of the Arabian Peninsula, i.e. *Cx. decens*, *Cx. duttoni*, *Cx. simpsoni* and *Cx. univittatus*. These species were only known to occur in Yemen [5, 108] until they were more recently found in the Asir Region [63], Jizan (Jazan) Region [18] and Najran Province [28], which border Yemen in the southwest corner of the KSA. The records of two of these species, *Cx. simpsoni* and *Cx. univittatus*, require confirmation because their identification was based on larvae and adults. They are only reliably distinguished from *Cx. sinaiticus* and *Cx. perexiguus*, respectively, based on the morphology of the male genitalia. Records of the two species in more northerly areas of the country are more doubtful.

Since *Culex salisburiensis* has been known to be present in Yemen since 1953 [108], there is no reason to doubt its occurrence in the adjacent Afrotropical area of the KSA [21, 67]. On the contrary, the reported finding of *Cx*. *wigglesworthi* in the Asir Region by Al Ashry et al. [67] is certainly a misidentification. The authors collected 4307 *Culex* larvae, two of which were identified as *Cx. nebulosus*, three as *Cx. perexiguus* and two as *Cx. wigglesworthi*. Larvae were identified using the keys of Hopkins [109], Mattingly & Knight [2] and Harbach [4, 5]. The identification of *Cx. nebulosus* and *Cx. perexiguus* may be correct as these two species are included in the keys of Mattingly & Knight (*Cx. nebulosus*) and Harbach (*Cx. nebulosus* and *Cx. perexiguus*). Use of the badly outdated keys of Hopkins [109] for African species published in 1952, leading to the identification of *Cx. wigglesworthi*, is problematic, nonetheless because it is a sub-Sharan species with closest records in southern Sudan, South Sudan and Kenya. Consequently, as it seems likely that the two larvae were misidentified specimens of either *Cx. perexiguus* or *Cx. univittatus*, *Cx. wigglesworthi* is excluded from the list of mosquito species known to occur in the KSA.

Alahmed [35], Alahmed et al. [36] and Khater et al. [7] recorded the presence of *Uranotaenia* (*Pseudoficalbia*) *unguiculata* in eastern and southwestern areas of the KSA. The authors, however, were apparently unaware of the earlier description of *Ur. unguiculata pefflyi* by Stone [69], and the recognized presence of only that form in eastern parts of the KSA [31]. Consequently, as it is unlikely that the typical form, *Ur. unguiculata unguiculata*, is present in the country, only the subspecies described and named by Stone [69] is included in the list.

## Conclusions

It is obvious from this study that researchers working in the KSA have generally been unaware of the limitations of available keys, have failed to critically evaluate published records and, at least in the case of *Culex*, have not confirmed identifications with correlated diagnostic features of the male genitalia. Future studies in the KSA must overcome these shortcomings by individually rearing larvae to obtain adults of both sexes for integrated morphological and molecular study, and use the resultant unequivocal molecular markers for the identification of larvae and adults collected in subsequent studies. Based on currently available information, it is concluded that 49 species of Culicidae, 18 anophelines and 31 culicines, are present in various regions of the KSA.

## Data Availability

Not applicable.
